# Assessment of Heat Shock Protein 70 Induction by Heat in Alfalfa Varieties and Constitutive Overexpression in Transgenic Plants

**DOI:** 10.1371/journal.pone.0126051

**Published:** 2015-05-07

**Authors:** Nicoletta Ferradini, Rina Iannacone, Stefano Capomaccio, Alessandra Metelli, Nadia Armentano, Lucia Semeraro, Francesco Cellini, Fabio Veronesi, Daniele Rosellini

**Affiliations:** 1 Department of Agricultural, Food and Environmental Sciences, University of Perugia, Perugia, Italy; 2 ALSIA- Research Center Metapontum Agrobios, S.S. Jonica 106 Km 448,2–75012 Metaponto, Italy; University of Tsukuba, JAPAN

## Abstract

Heat shock proteins (HSPs) are molecular chaperones involved in many cellular functions. It has been shown that mammalian cytosolic HSP70 binds antigenic peptides mediating the activation of the immune system, and that it plays a determining role in tumour immunogenicity. This suggests that HSP70 may be used for the production of conjugated vaccines. Human and plant HSPs share high sequence similarity and some important biological functions *in vitro*. In addition, plant HSPs have no endotoxic side effects. Extraction of HSP70 from plants for use as vaccine adjuvant requires enhancing its concentration in plant tissues. In this work, we explored the possibility to produce HSP70 in both transgenic and non-transgenic plants, using alfalfa as a model species. First, a transcriptional analysis of a constitutive and an inducible HSP70 genes was conducted in *Arabidopsis thaliana*. Then the coding sequence of the inducible form was cloned and introduced into alfalfa by *Agrobacterium*-mediated transformation, and the accumulation of the protein in leaf tissue of transgenic plants was demonstrated. We also tested diverse alfalfa varieties for heat-inducible expression of endogenous HSP70, revealing variety-specific responses to heat shock.

## Introduction

Heat shock proteins (HSPs) are a class of proteins conserved in all living organisms and involved in basic cellular functions such as homeostasis, folding of nascent proteins, refolding of denatured proteins, preventing protein aggregation and assisting protein transport across membranes [1]. These functions earned them the name “molecular chaperones”.

Some HSPs are expressed constitutively in all cells (cognate proteins or HSC) whereas others are transiently activated in response to several stimuli such as heat, drought, cold, chemical and oxidative or physiological stress and pathogen attack (inducible HSPs).

In plants, inducible HSPs are transiently activated after heat shock (HS) and their expression level remains high up to few hours after induction; this response is dependent on the genotype and the environmental conditions [2].

HSPs are classified into several families according their molecular size [3]. They are located in all cellular and subcellular compartments (nucleus, membrane, cytosol, endoplasmic reticulum, mitochondria, lysosome).

The 70 kDa HSP superfamily constitute a large and highly conserved family of chaperones in both prokaryotes (DnaK) and eukaryotes (HSP70) [4]. The *Arabidopsis* genome encodes 18 members of the HSP70 superfamily including 14 in the DnaK subfamily and 4 in the Hsp110/SSE subfamily [5]. It was hypothesized that stress-induced accumulation of inducible HSP70 accelerates cellular recovery and protects cells from several stresses *in vivo* and *in vitro* [6, 7].

In the last decade, HSP70 and HSP90 have been assigned a new role of danger signals and potent activators of the immune system [8–10]. It has been shown that these molecules are able to induce the production of proinflammatory cytokines by the monocyte-macrophage system and the activation of antigen-presenting cells (APCs), such as dendritic cells. Mammalian cytosolic HSP70 and HSP90 bind antigenic peptides generated in cells and these complexes (HSP-PC) are taken up by antigen presentation cells via α_2_-macroglobulin receptor (CD91)-mediated endocytosis, resulting in representation by the Major Histocompatibility Complex (MHC) class I molecules [11]. The capability of HSPs, particularly HSP70 and HSP90, to function as molecular carriers for antigenic determinants suggests the possible application of these molecules for the production of conjugated vaccines [12].

Several studies in experimental models demonstrated that HSP70 plays a determining role in tumour immunogenicity and is responsible for the rejection of tumours by the immune system. Vaccination with tumour-derived HSPs was shown to be successful against several tumours, induced and spontaneous, of different histological origins [13–15].

Recently, it has been shown that chromatographic purification of HSP70 is an effective way to extract antigens overexpressed in plants, thanks to spontaneous formation of HSP70-polypeptide complexes. This was demonstrated using *Nicotiana benthamiana* plants expressing an influenza virus A nucleoprotein after agroinfiltration: the extracts were able to prime both T cell-mediated and humoral immune responses without the need of adjuvant co-delivery [16, 17].

Human and plant HSPs share high sequence similarity and they show the conservation of some important biological functions *in vitro*. In addition, plant HSPs have no endotoxic side effects and mediate activation of the mammalian immune system [18].

Therefore, extraction of HSP70 from plants may be economically interesting [19]. However, the natural concentration of the HSP70 protein in plant tissues is not sufficient for industrial production.

Two alternative ways could be proposed to enhance HSP70 concentration in plant tissues. The first is subjecting plants to heat shock, so that concentration of the inducible form of HSP70 increases, and harvesting the plants when protein concentration is maximum. Heat shock can be administered in the field by means of heat producing machines. The second is overexpressing HSP70 in plants by genetic transformation.

In the past years, many recombinant proteins were experimentally produced in plants since the feasibility to produce mammalian pharmaceutical proteins was shown [20, 21]. In this work, we explored the possibility to accumulate one of the inducible forms of HSP70, that was previously demonstrated to be upregulated upon stress in *Arabidopsis* [22], in both transgenic and non transgenic plants, using alfalfa as a model species. Alfalfa is a leguminous forage crop that produces a large amount of biomass thanks to its perenniality, is beneficial for the environment and is not directly used for human consumption; these features make it an interesting candidate for the production of useful proteins. First, a transcriptional analysis of a constitutive and an inducible *HSP70* genes was conducted in *Arabidopsis thaliana*. Then the coding sequence of the inducible form was cloned and introduced into alfalfa by genetic transformation via *Agrobacterium tumefaciens*, and the accumulation of the protein in leaf tissue was assessed. Finally, we tested diverse alfalfa varieties for heat-inducible expression of endogenous HSP70.

## Materials and Methods

### *Arabidopsis HSP70* RNA isolation and expression analysis

*Arabidopsis* seedlings (Ecotype Columbia, 1 month old) were subjected to heat treatments in a laboratory oven with temperatures ranging from 40°C to 60°C followed by a recovery phase at room temperature, as described in Table 1. Green tissues were immediately frozen in liquid nitrogen after harvesting. Total RNA was extracted using the ToTALLY RNA Total RNA Isolation Kit (Ambion/Life Technologies, Grand Island, NY). Total RNA concentration was measured using the NanoDrop 1000 spectrophotometer (Thermo Scientific, USA) and the integrity was checked by agarose gel analysis. RNA was treated with DNase I (Ambion, Norwalk, CT, USA) and double stranded cDNA was prepared using 2 μg of total RNA, oligo-(dT)_18_ primer and the ThermoScript Reverse Transcriptase kit (Life-Technology) following the manufacturer instructions.

**Table 1:** Temperature treatments applied to Arabidopsis plants.

Treatment	Temperature (°C)	Exposure time(min)	Recovery after heat shock (min)
**A**	20 (not induced)	-	-
**B**	40	30	0
**C**	40	60	0
**D**	40	90	0
**E**	50	60	30
**F**	50	60	60
**G**	60	15	0
**H**	60	15	30
**I**	60	15	60
**L**	4	2880 (48 h)	-

The expression level of *HSC70* and *HSP70* in the heat treated samples was verified by real-time quantitative reverse transcription PCR (RT-qPCR) with oligonucleotides designed on the basis of the AtHSC70 (AL162971) and AtHSP70 (AJ002551) sequences [22], using the Beacon designer 2.0 software (BIO-RAD) and the iCycler iQ Multicolor Real Time PCR detection system (BIO-RAD) with the Quantitect probe PCR kit (Qiagen).

Primers and TaqMan probes were as follows: HSC70: probe FAM-5’TGCGAATCATCAACGAGCCTACAGCC-TAMRA, forward primer 5’TGGTGTCATCGCTGGTTTGAA, reverse primer: 5’ATTCTTCTCTCCAACGCTGGTAG; HSP70: Probe FAM-5’AACTGCTGCTGCTATTGCTTACGGTCTT-TAMRA, forward primer 5’GTCTCAACGTGATGCGTATCATC, reverse primer 5’AGTACCACCTCCCAAATCAAAGAT.

The 18S rRNA was used as housekeeping transcript to normalize the expression of the target genes. Primers and TaqMan probes for 18s were as follows: Probe FAM-5’AAGGCAGCAGGCGCGCAAA-TAMRA, forward primer 5’GAAACGGCTACCACATCCAAG; reverse primer 5’CCCCGTGTTAGGATTGGGT.

The PCR efficiency was evaluated using the standard curves obtained by the means of a series of five cDNA dilutions for target and housekeeping genes. Each sample was replicated 3 times. The normalized expression ratio was calculated using the 2^−ΔΔCT^ method. (User Bulletin #2, ABI PRISM 7700 Sequence Detection System, Relative quantitation of gene expression, 2001, AB Applied Biosystems).

### Cloning of the inducible *HSP70* open reading frame

**Fig 1:**
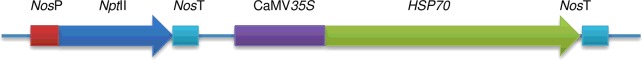
T-DNA of binary vector pJazz-*HSP70* used for alfalfa transformation.

The cDNA obtained from *Arabidopsis* leaves treated at 40°C for 30 min (treatment B) was used as template for the amplification of the full length inducible *HSP70* ORF using the proofreading Expand PCR System (Roche). Primers were as follows: AtHSP70F: 5'TAATGGCGGGTAAGGTGAA; AtHSP70R: 5'GCCAAAAGGCTTAATCAACTTC. The amplified product was cloned using the TOPO TA Cloning Kit (Life-Technology) and sequenced. The cloned fragment was then amplified by PCR with primers containing the *Xba*I and *Bam*HI recognition sequences to the 5’ and 3’ end respectively (forward primer: 5’tctagaATGGCGGGTAAAGG; reverse primer: 5’ggatccGCCAAAAGGCTTA, restriction sites in small case) and the gene was cloned under the transcriptional control of the *CaMV35S* promoter and the *Nos* terminator in a Bin19-derived [23] plant transformation vector, thus obtaining the pJAZZ-HSP70 vector (Fig 1). The correctness of the *HSP70* ORF was checked by sequencing. The plasmid was introduced into *A*. *tumefaciens* strains LBA4404 and AGL1 by electroporation

### Heat shock treatment of alfalfa varieties

Twelve alfalfa varieties (Table 2) were selected based on a *M*. *sativa* variety recommendation lists for Italy provided by the Animal Production Research Centre (CRPA, Reggio Emilia, Italy) [24]. Top performing varieties from different Italian regions were chosen. Seeds of a North African germplasm and a Canadian variety were obtained from Bernadette Julier (INRA, Lusignan, France) and from Real Michaud (Agriculture and Agri-Food, Canada).

**Table 2:** Alfalfa varieties used in this work.

Variety	Registration year	Breeder	Country of origin	Longevity^(a)^	Productivity^(a)^
**Arpege**	2003	Limagrain Italy	Italy	-	-
**Azzurra**	2003	S.I.S.—Società Italiana Sementi	Italy	Good	High
**Casalina**	2004	University of Perugia	Italy	-	-
**Classe**	1997	CO.NA.SE. Consorzio Nazionale Sementi	Italy	Good	Medium
**Equipe**	1978	Istituto Sperimentale Colture Foraggere (LO)	Italy	Good	Medium
**Garisenda**	1976	S.I.S.—Società Italiana Sementi	Italy	High	High
**Iside**	1994	Istituto Sperimentale Colture Foraggere (LO)	Italy	Good	Good
**Memont**	-	ARSIA Abruzzo, Italy	Italy	-	-
**PR57N02**	1999	Pioneer Hi-Bred—Usa	Italy	Good	High
**Zenith**	2000	Florimond Desprez (France)	France	Good	Medium
**Caribou**	-	Provided by Real Michaud, Agriculture Canada	Canada	-	-
**Moroccan**	-	Provided by Bernadette Julier, INRA, France	Morocco	-	-

^**(a**)^ As reported by (24)

Seeds of each variety were scarified and sown in jiffy pots filled with growth substrate and placed on a 7 cm layer of the same substrate in a plastic flat. The substrate was made of soil, sand and peat moss (3:1:1 in volume) mixture. Two flats, each consisting of 80 plants, were reared per variety. After three weeks of growth at the experimental farm of the University of Perugia, one flat per variety was placed for 10 min at 60°C in a phytotron. After 30 min of recovery at room temperature, leaf samples (the apical three fully expanded leaves of each plant) were collected and immediately frozen in liquid nitrogen. At the same time, leaf samples were collected from the control flat kept at room temperature. Two replications per variety per treatment were obtained by dividing the plants of each flat into two blocks.

### Alfalfa Transformation with the *HSP70* Gene

#### Plant Transformation

The alfalfa genotype RSY1, selected for high somatic embryogenesis capability from the Regen-SY germplasm [25, 26] was used. Young, fully expanded leaves collected from 4 to 6 week-old greenhouse grown plants were surface sterilized with commercial bleach (0.5%). and used for *Agrobacterium tumefaciens*-mediated transformation.

Alfalfa transformation was performed with pJazz-*HSP70* binary vector carried by *A*. *tumefaciens* LBA4404 and AGL1 strains. Liquid culture, infection, and co-culture of alfalfa leaf explants with LBA4404 strain were carried out as previously described [27] whereas AGL1 transformation was performed as described [28]. Both transformations were performed with kanamycin selection, 50 mg l^-1^. Leaf explants not treated with *Agrobacterium* were cultured in selective conditions (negative controls) and in non-selective conditions (positive control). Green and well conformed somatic embryos from *Agrobacterium*-treated and from the positive control plates were picked avoiding to pick multiple embryos from the same transformation event, and directly converted into plants as described by [28]. The plants were transferred to sterile soil in pots and reared for about a month, until they flowered.

#### PCR analysis

The genomic DNA was extracted from young leaves of putative transgenic plants using the GeneElute Plant Genomic DNA Miniprep Kit (Sigma) according to the manufacturer’s protocol. PCR screening of transgenic plants was carried out for the *HSP70* gene using the primers pair *HSP70 screening FOR* (5’TGGGAATCAACTGGCTGAGG) and *HSP70 screening REV* (5’GCAAGACCGGCAACAGGATT) designed on the coding sequence of the HSP70 gene and on the *nos* terminator, respectively. For *npt*II gene the primer pair *NPTII real time FOR* (5’ACTGTTCGCCAGGCTCAAGG) and *NPTII real time REV* (5’CCGCCAAGCTCTTCAGCAAT), designed on the coding sequence of the gene, were used. All the PCR amplifications were performed using 1x Buffer, 1.5 mM MgCl_2_, 0.2 mM dNTPs, 0.4 μM primers, 1U Taq (Sigma), in a final volume of 25 μl. Thermal cycling for *HSP70* screening FOR/REV was: 94°C for 1 min, 35 cycles at 94°C for 20 s, 65°C for 20 s, and 72°C for 20 s, final extension at 72°C for 10 min. For *HSP70 screening FOR/REV* the annealing temperature was 64°C and for *NPTII real time FOR/REV* was of 64°C. Electrophoresis in 2% agarose gel was carried out to detect the amplicons.

#### Reverse-transcriptase PCR

Total RNA was extracted using the Spectrum Plant Total RNA Kit (Sigma) according to the manufacturer’s instructions. To remove contaminating genomic DNA, the RNA was treated with DNase I (Ambion, Norwalk, CT, USA). First strand cDNA was synthesized from 2 μg of total RNA using SuperScriptII Reverse Transcriptase (Invitrogen, http://www.invitrogen.com), according to the manufacturer’s protocol. A qualitative RT-PCR was performed using 5 μl of cDNA and the specific primer pair *HSP70* screening FOR/REV. RT-PCR amplicons were subjected to electrophoresis in a 2% agarose gel.

#### Segregation analysis of the transgenes

To confirm transgene integration and to estimate the number of integration sites, the three transgenic plants (pJazz 2, 3 and 6) were hand-crossed as females without emasculation with an unrelated non-transgenic alfalfa plant from the Italian variety Classe, as the pollen donor. After about a month, seeds were harvested, and 40 T1 seeds per progeny were scarified and sown in Petri dishes containing wet filter paper; seedlings were then transplanted to pots, and leaf samples were taken after 2 weeks. DNA was extracted from leaf tissue of each progeny plant using the DNeasy 96 Plant Kit (Qiagen) according to the manufacturer’s instructions. The presence of the HSP70 gene in the progeny of the three transgenic plants was assessed by PCR using the primer pair *CAMV35S-END* (5’CGCACAATCCCACTATCCTT) and *HSP70-REV* (5’GCCAACACTCGACGCCTTCT). PCR amplification was performed using 1X Buffer, 1.5 mM MgCl_2_, 0.2 mM dNTPs, 0.4 μM primers, 1U Taq (Sigma), final volume 25μl. Thermal cycling was: 94°C for 1 min, 35 cycles at 94°C for 20 s, 65°C for 20 s, and 72°C for 20 s, final extension at 72°C for 10 min. Progeny plants were scored positive or negative for the two genes after agarose gel electrophoresis.

#### Protein extraction and Western blot analysis

Total soluble proteins were extracted from leaf samples of heat treated and control alfalfa varieties (WT) and from alfalfa transgenic plants. The leaves were ground to a fine powder in liquid nitrogen and the powder was suspended in water. The homogenates were centrifuged twice at 10,000 RCF for 20 min at 4°C and the soluble proteins were quantified in the supernatant. For each treatment and variety, equal amounts of protein from each of the two replicates were mixed. Total soluble protein concentration was determined as described in [29] using bovine serum albumin (BSA) as standard. Equal amounts of proteins per sample (20 μg) were separated by 10% SDS PAGE and transferred to a nitrocellulose membrane (PROTRAN, Shleicher & Schuell) by a Trans-Blot Semy-dry (BioRad) containing transfer buffer (25 mM Tris, 192 mM glycine and 20% (v/v) methanol, pH 8.3). Protein electrophoresis was performed in triplicate.

Western analysis was performed according to standard procedures using as primary antibody a polyclonal rabbit HSP70 antibody (obtained against the AtHSP70 protein) for alfalfa proteins (dilution 1:10000). A peroxidase-conjugated anti-rabbit IgG diluted 1:10000 was used as secondary antibody (Sigma).

Hybridized blots were visualized with the SuperSignal West Pico HRP Substrate Kit (Thermo scientific, Pierce protein RP, Rockford, IL USA) and exposed with Kodak AR films (Kodak, Rochester, New York, USA). Quantification of the HSP70 protein was carried out using the recombinant AtHSP70 produced in *E*. *coli* BL21 cells as standard; 0.2 μg and 0.4 μg were loaded on SDS-PAGE along with plant samples.

#### Data analysis

HSP70 protein band intensity was measured on alfalfa western blot films using the ImageJ software. The data were then analyzed by means of the ANOVA procedure of SAS (Statistical Analysis System, Cary, NC, USA). Two-way ANOVA was performed using the treatments (heat shock *vs* control) and cultivars as the classification variables. The segregation ratios of PCR-positive and negative plants in the T1 progenies of pJazz 2, 3 and 6 plants were compared with Mendelian expectations for one and two loci by X^2^ analysis.

## Results

### Transcription level of *HSC* and *HSP* genes in *Arabidopsis*

**Fig 2:**
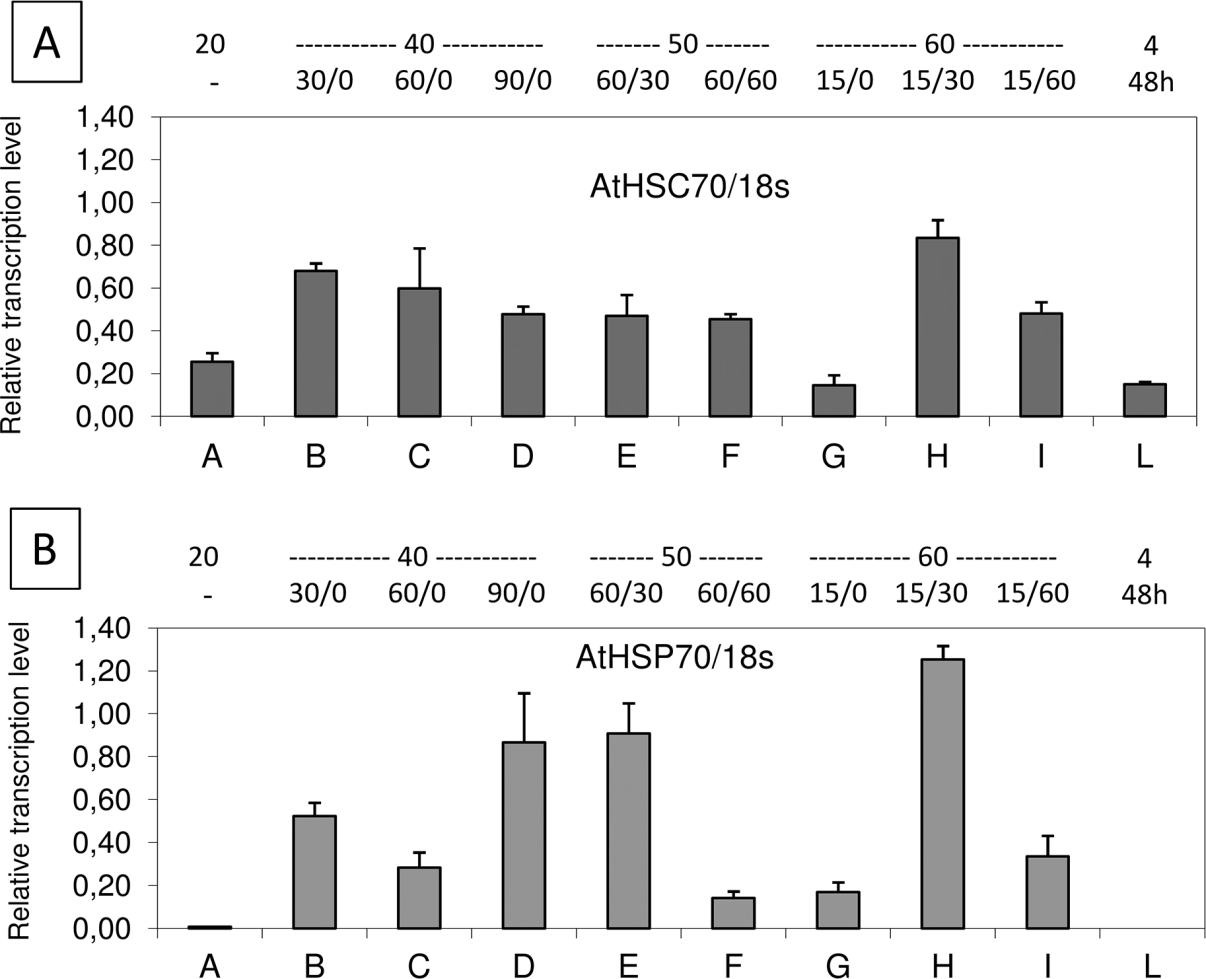
RT-qPCR analysis of *HSC70* (A) and *HSP70* (B) transcript levels in *Arabidopsis* plants subjected to different heat stress treatments, as outlined in Table 1. Numbers above the graphs are temperature treatments (°C, first line), and duration of treatments/duration of recovery (minutes, second line).

The accumulation of *HSC70* and *HSP70* transcripts was assessed in leaves of *Arabidopsis* plants treated at different temperatures as outlined in Table 1. The two genes were differently modulated upon stress (Fig 2). *HSC70* was confirmed to be constitutively expressed [22], with a basal level of expression in non-treated plants and an increase following HS. We observed an increase of the cDNA level already after 30 min at 40°C (treatment B) and no further increase with longer exposures (treatments C and D). With the treatments at 50°C (E, F) the mRNA level did not further increase. After treatment at 60°C, the mRNA levels increased only if there was a recovery of at least 30 min (G-I).

As regards *HSP70*, as expected, we did not observe detectable expression at 20°C, while transcription raised rapidly at 40°C. Treatment at 50°C for 60 min (E-F) lead to high gene expression if followed by 30 min recovery. The highest transcript level was observed after treatment at 60°C for 15 min and a recovery of 30 min (H). Plants kept at 4°C did not contain detectable *HSP70* mRNA.

### HSP70 accumulation in alfalfa varieties

**Fig 3:**
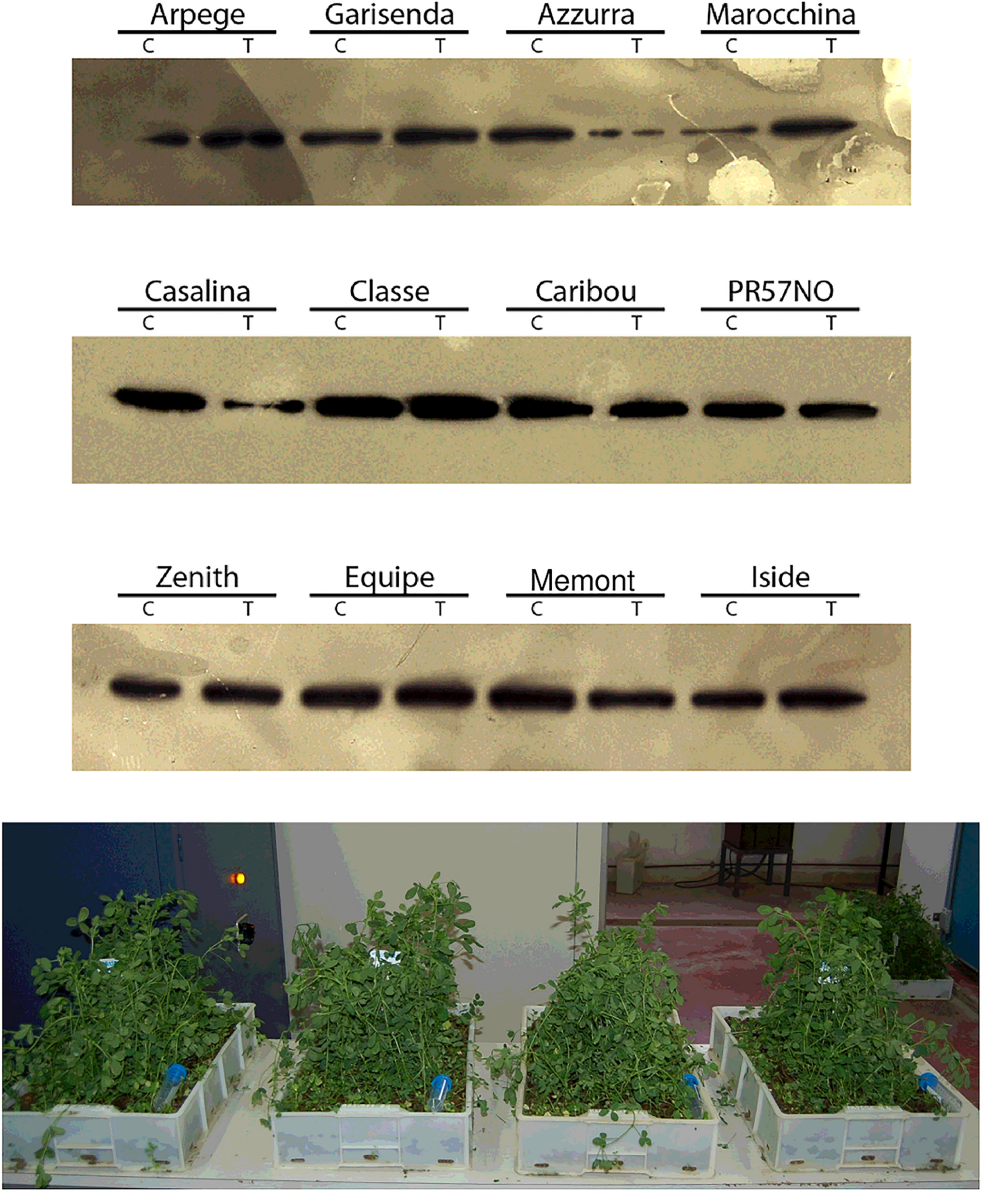
Above: Example of western blot for HSP70 detection in alfalfa varieties subjected (T) or not (C) to heat shock at 60°C. The protein amounts in this replicate does not necessarily reflect the average data from the three replicates presented in Table 3 (details are provided in the text). Below: plants of three varieties during the recovery period after heat stress, showing slightly different wilting degrees.

Western blot analysis allowed to detect the HSP70 protein in all the alfalfa varieties in both control and heat stressed leaf samples (example in Fig 3).

The ANOVA showed that the HS treatment and the interaction between treatment and cultivars did not significantly affect HSP70 concentration (*P* = 0.190 and 0.151, respectively). The cultivars differed at *P* = 0.064. There was a three-fold range of HSP70 concentration among cultivars, with Garisenda and PRS7NO producing the highest, and Casalina and Zenith the lowest amount (Table 3).

**Table 3:** Relative content of the HSP70 protein in alfalfa varieties subjected or not to heat shock (HS).

Variety	HSP70 content				
	HS	Control	Mean		Variation HS/Control %
**Garisenda**	31.44	33.42	32.43	A	-6
**PR57N02**	15.25	37.68	26.47	AB	-60
**Moroccan**	20.40	28.19	24.30	ABC	-28
**Caribou**	25.06	23.26	24.16	ABC	+8
**Iside**	28.21	19.37	23.79	ABC	+46
**Azzurra**	10.73	33.84	22.29	ABC	-68
**Equipe**	19.12	21.63	20.38	ABC	-12
**Arpege**	19.49	12.70	16.10	ABC	+53
**Memont**	16.82	20.59	18.71	BC	-18
**Classe**	8.43	18.12	13.28	C	-54
**Zenith**	14.91	9.78	12.35	C	+53
**Casalina**	15.18	8.25	11.72	C	+84

The HSP70 protein concentration was measured from western blot bands by the ImageJ software. Means followed by different letters are significantly different according to the Duncan multiple range test (n = 3).

The effect of HS varied between cultivars (Table 3), and was not always consistent between replications. Casalina, Arpege, Zenith and Iside appeared to respond positively to HS, with an average increase in HSP70 accumulation of 84, 53, 53, and 46%, respectively (Table 3); Azzurra, PRS7NO, Classe and Moroccan appeared to respond to HS with a reduced HSP70 concentration.

We observed that the varieties in which HS induced HSP70 accumulation quickly recovered after stress, whereas those showing reduction of HSP70 did not completely recover, and remained visibly wilted; the rest of the cultivars showed different responses between replications (Fig 3).

### Alfalfa transformation and overexpression of *AtHSP70* gene

From 200 leaf explants infected with the *Agrobacterium* strain LBA4404 harboring the pJazz-35s-*HSP70* binary vector, no kanamycin resistant somatic embryos were produced, whereas from the infection of 150 explants with the more virulent AGL1 strain harboring the same plasmid, six somatic embryos were recovered, from which three kanamycin resistant plants were obtained (hereafter named Jazz2, Jazz3 and Jazz6). The three putative transgenic plants were analyzed by PCR and all of them were positive for the *HSP70* gene using the primer pair *HSP70* screening FOR/REV (not shown). The transformation efficiency was therefore extremely low, much lower than those generally observed for both *Agrobacterium* strains in our lab, and in general for alfalfa transformation.

**Fig 4:**
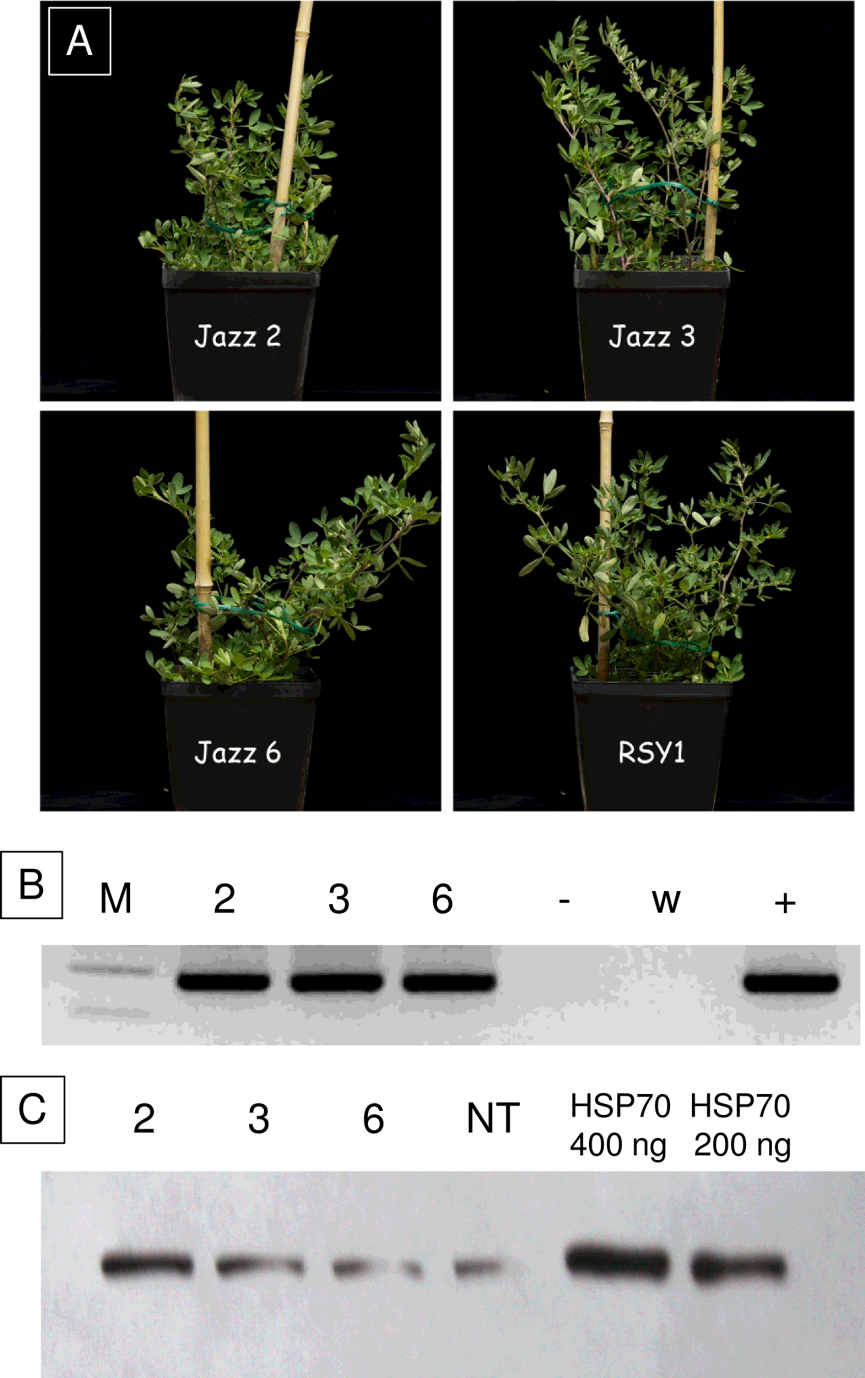
Analysis of the three *HSP70*-transgenic plants. **A**: Phenotypes of the three transgenic plants compared with the non-transgenic control (RSY1) during vegetative growth. **B:** Result of RT-PCR amplification of 260 bp of the *HSP70* mRNA, with the Primers *HSP70 Screening FOR* and *REV*. 2, 3, 6: transgenic plants Jazz2, 3, 6, respectively;-: negative control (c-DNA of RSY1); w: water (no DNA); **+**: positive control (plasmid pJAZZ-*HSP70*). M: molecular weight marker (GeneRuler DNA Ladder Mix, Fermentas); the two visible bands of the ladder are 300 and 200 bp. **C:** Western blot analysis of the HSP70 protein. The two rightmost lanes contain the HSP70 control protein for quantification.

No obvious phenotypic alteration was observed in the putative transgenic alfalfa plants under growth room conditions (Fig 4A), and the plants flowered and set seed normally. RT-PCR analysis confirmed the presence of the *HSP70* transcript in the three transgenic plants, whereas no amplicon was generated in the non-transformed control (Fig 4B).

To confirm transgene integration, the T1 progenies obtained from the three transgenic plants were screened by PCR for segregation of the *HSP70* gene. All the progenies showed transgene segregation and the estimated number of loci was one in Jazz 3 and Jazz 6, and one or two in Jazz 2 (Table 4).

**Table 4:** Transgene segregation in the T1 progenies of the three HSP70 transgenic plants.

Event	T1 progenies	PCR +	PCR -	Χ^2^	
				**1 locus (1:1)**	**2 loci (3:1)**
**Jazz 2**	32	21	11	3.125 NS	1.500 NS
**Jazz 3**	32	15	17	0.125 NS	13.500 **
**Jazz 6**	32	13	19	1.125 NS	20.166 **

X^2^ for *P* = 0.01 and 1 df is 6.63; X^2^ for *P* = 0.05 and 1 df is 3.84. NS: not significant.

**: significant at *P*<0.01

Western analysis detected a HSP70-specific signal of the expected molecular weight in all the transformed plants (Fig 4C). The negative control RSY1 also showed a HSP70 band, as expected, produced by the endogenous HSP70 protein. The quantification of the HSP70 protein was carried out relative to the standard, loaded in the gel (200 and 400 ng, Fig 4C). The amounts of protein ranged between 0.66 to 2,25 μg, corresponding to 0,144–0,451% of total soluble protein (Table 5). The highest percentage of soluble protein was produced by Jazz 2, in which HSP70 was 3.4-fold concentrated with respect to the non-transgenic control.

**Table 5:** Amount of the HSP70 protein in the three transgenic plants and in the non- transgenic control as estimated by western blot.

Transgenic plant	Protein per mg leaf tissue, μg	Soluble protein (%)
**Jazz 2**	2.25	0.451
**Jazz 3**	1.10	0.220
**Jazz 6**	0.72	0.144
**RSY**	0.66	0.131

## Discussion

### Expression analyses of cognate and inducible HSP70 in *Arabidopsis*

A transcriptional analysis of the *HSP70* and *HSC70* genes in *Arabidopsis* was performed in order to confirm the heat inducibility of *HSP70*. By comparing the mRNA level of the two genes we confirmed that *HSP70* is more responsive to HS than *HSC70*. In fact, we observed the presence of the *HSC70* mRNA in all plants, including those kept at room temperature. Nevertheless, the *HSC70* mRNA level was very low when plants were not allowed to recover after heat stress or when the plants were kept at 4°C for 48 hours. In the case of *HSP70*, we did not detect the specific RNA in tissues kept at room temperature, confirming previous findings [20]; the highest RNA level was detected when plants were allowed to recover for 30 min after HS (50°C and 60°C) while we observed a lower *HSP70* RNA when tissues were harvested immediately after HS or when the recovery period was longer. This can be explained by a temporary repression of RNA synthesis during heat treatments at 50 and 60°C, and by a rapid RNA turnover. Generally, 30 min recovery favoured maximum mRNA accumulation after HS.

We did not observe *HSP70* RNA expression in samples kept at 4°C for 48 hours, indicating that the gene is not cold-responsive.

### Assessment of alfalfa varieties for heat-induced HSP70 production

Among the 12 varieties tested, Iside, Casalina, Zenith and, to a lower level, Arpege and Caribou, showed an increase of HSP70 concentration as a consequence of HS. Other varieties like Azzurra, Classe and PRS7NO, showed an opposite response, with a marked reduction of protein accumulation. The fact that the first group of varieties showed complete recovery after stress, whereas the second group did not, indicates that the ability to rapidly accumulate HSP70 may provide protection from cellular damage induced by the heat shock. Reduction of HSP70 concentration in the second group of varieties as a result of HS, may be a cause as well as an effect of HS. It is possible that lower temperature and/or shorter duration of the heat treatment would have resulted in different varietal responses.

Interestingly, the ‘responsive’ varieties Arpege, Casalina, and Zenith showed low level of HSP70 under normal condition, whereas ‘non responsive’ varieties had a relatively high concentration of the protein. The varieties from extreme climates, Moroccan, adapted to north Africa, and Caribou, adapted to Canada, did not differ for HSP70 concentration, both in control and stress conditions.

### Assessment of transgenic alfalfa for HSP70 production

Unexpectedly, the transformation efficiency attained in this work was very low. In previous studies, the RSY1 genotype was easily and efficiently transformed with the same *Agrobacterium* strains [27, 28, 30]. Previous attempts of transforming alfalfa with the same construct had failed while alfalfa transgenic plants were obtained using non-constitutive promoters (Iannacone et al. unpublished). This may be explained by hypothesizing that the constitutive overexpression of the HSP70 protein impairs the regeneration of somatic embryos. If this is the case, the three regenerated plants may derive from transformation events exhibiting relatively low protein accumulation. Low transformation efficiencies were also observed [31] when transforming *Arabidopsis* with a vector for constitutive overexpression of Hsc70-1.

A detrimental effect of the overexpression of the bacterial DnaK protein was observed in *Escherichia coli* [32]: inhibition of cell septation, alteration in the growth rate and reduction of cell viability. Some of these deleterious effects were completely or partially suppressed by co-overexpression of the DnaJ protein (belonging to the hsp40 class of proteins), indicating that DnaK toxicity could depend on unbalanced expression of the two proteins. A similar result was obtained in *Synechococcus* strain PCC7942 [33].

Eukaryotic HSP70 proteins have a 40–60% nucleotide identity with *E*. *coli DnaK* and also interact with other proteins [34]. In fact, HSP70 proteins are regulated by several co-chaperones, in particular HSP40, which stimulates HSP70 cytosolic ATPase activity [35, 36]. It is possible that, as in prokaryotic organisms, the overexpression of HSP70 alone, without co-chaperons, has detrimental effects in plant cells. Overexpression of Hsc70-1 caused reduced shoot and root meristem activity and dwarfism [4].

In this experiment, the expression of HSP70 in alfalfa did not have any obvious detrimental effect as regards growth, development and fertility. We did not find any previous report of the over-expression of this HSP70 form in plants.

## Conclusion

In the last decade, plants have been the object of extensive studies in order to use them as an alternative to bacterial, yeast, insect or mammal cell cultures for the production of valuable proteins for pharmaceutical use or human consumption. The use of plants as bioreactors for the production of proteins for therapeutic purposes has many advantages like lower production costs, the absence of humans pathogenic contaminants such as microbial toxins or oncogenic compounds [37]. The commercial production of the pharmaceutical protein taliglucerase alfa for Gaucher disease treatment in carrot cells started recently [38].

Our results indicate that alfalfa can be suitable for the production of HSP70 protein. On one hand, we obtained indications that variability exists among varieties for both uninduced and heat-induced HSP70 production. Likely, genotypes to be used as green bioreactors may be obtained by further screening or plant breeding programs.

Transgenic alfalfa overexpressing HSP70 may also allow to produce the protein, without the necessity of exposing the plants to thermal stress; in fact, the heat treatment in the field has considerable costs and may not be always effective due to technical difficulty and environmental interactions. For the transgenic approach, however, the observed low transformation efficiency will need to be addressed, and higher accumulation would be advantageous. Both objectives may be pursued by substituting CaMV35S with a promoter that is activated by chemical or physical stimuli or after leaf detachment and is stronger in green tissues. Plastid transformation may also be a good strategy to obtain higher concentration of the protein in leaves.
